# Supplementary material for: Genetic Association between Inflammatory-Related Polymorphism in STAT3, IL-1β, IL-6, TNF-α and Idiopathic Recurrent Implantation Failure
Source: Genes (Basel). 2023 Aug 5;14(8):1588. doi: 10.3390/genes14081588 (PMC10454471; doi:10.3390/genes14081588)
Supplement: Supplementary file 1 [file genes-14-01588-s001.zip › Supplentary Table S1.pdf]

Supplementary Table S1. Statistical power of genetic association of less than 0.05 *p*-value in Table 2

Characteristic	AOR (95% CI)	Statistical power (%)
STAT3 rs1053004 AG	0.623 (0.409-0.947)	68.1
STAT3 rs1053004 GG	0.513 (0.269-0.978)	86.7
STAT3 rs1053004 Dominant	0.601 (0.406-0.889)	76.5
IL-6 rs1800796 GG	2.472 (1.083-5.645)	78.3
IL-6 rs1800796 Recessive	2.374 (1.053-5.350)	63.0
TNF-α rs1800629 GA	2.127 (1.200-3.769)	77.2
TNF-α rs1800629 Dominant	2.198 (1.246-3.878)	80.8

AOR, adjusted odds ratio.
