# Supplementary figures and images for: Genetic Association between Inflammatory-Related Polymorphism in STAT3, IL-1β, IL-6, TNF-α and Idiopathic Recurrent Implantation Failure
Source: Genes (Basel). 2023 Aug 5;14(8):1588. doi: 10.3390/genes14081588 (PMC10454471; doi:10.3390/genes14081588)

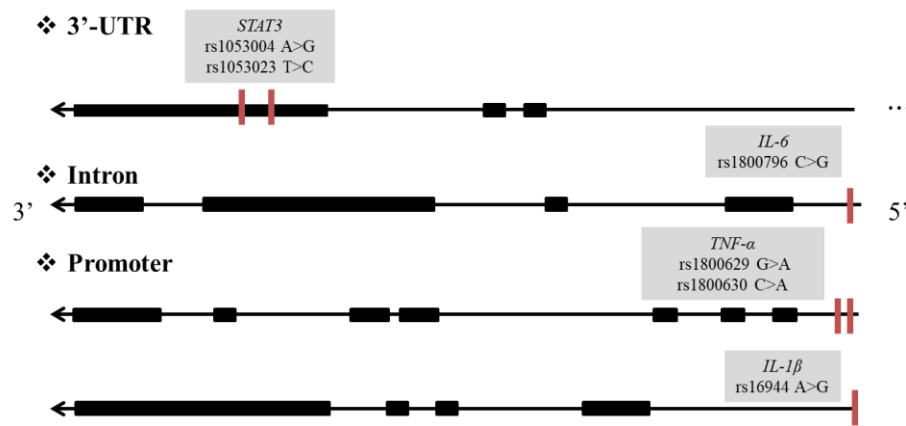

Supplementary Figure S1. Location of investigated polymorphisms

Supplement: Supplementary file 1 [file genes-14-01588-s001.zip › Supplementary Figure S1.pdf]
